# Comparative Study of Raw and HMDS-Treated Pigment-Rich Agro-Industrial By-Products as Functional Fillers in PDMS Composites

**DOI:** 10.3390/molecules31142546

**Published:** 2026-07-22

**Authors:** Khadija Ramzan, Sana Ullah, Sajjad Ahmad, Mudassar Hussain, Syeda Hijab Zehra, Aiste Balciunaitiene, Pranas Viskelis, Jonas Viskelis

**Affiliations:** 1 Lithuanian Research Centre for Agriculture and Forestry, Institute of Horticulture, Kaunas Str. 30, Kaunas District, 54333 Babtai, Lithuania; sana.ullah@lammc.lt (S.U.); mudassar.hussain@lammc.lt (M.H.); hijab.zehra@lammc.lt (S.H.Z.); aiste.balciunaitiene@lammc.lt (A.B.); pranas.viskelis@lammc.lt (P.V.); 2 The Sustainability and Health Research Hub (SHRH), School of Food Science & Environmental Health, Technological University Dublin-City Campus, Grangegorman Lower, GW212-35 Dublin 07, Ireland; sajjad.ahmad@tudublin.ie

**Keywords:** polydimethylsiloxane, surface modification, functional fillers, sustainable materials, agro-industrial waste

## Abstract

Agro-industrial by-products from beetroot, raspberry, sea buckthorn, and shadbush are valuable sources of natural pigments and renewable filler materials, but their hydrophilic surfaces limit compatibility with hydrophobic polydimethylsiloxane (PDMS) matrices. This study evaluated the effect of catalyst-free vapor-phase hexamethyldisilazane (HMDS) treatment of pigment-rich powders and their incorporation into PDMS composites at 5 and 20 wt.% filler loadings. Fourier-transform infrared (FTIR) spectroscopy, scanning electron microscopy, and contact angle measurements were used to characterize surface modifications. Mechanical behavior under puncture loading was evaluated using texture analysis. FTIR confirmed successful silylation through the reduction in hydroxyl groups and the emergence of silicon-containing functionalities. Treated fillers exhibited rougher, more irregular surfaces, which suggested improved filler dispersion and interfacial compatibility within the PDMS matrix. Modified composites showed enhanced hydrophobicity, with contact angles up to 102.84°. Composites containing 5 wt.% HMDS-treated raspberry filler demonstrated the highest puncture resistance and elasticity, indicating improved interfacial interactions. In contrast, 20 wt.% filler loading generally reduced puncture resistance and elasticity, possibly due to increased particle agglomeration and reduced matrix continuity, as suggested by the SEM observations, whereas shadbush-filled composites showed decreased performance after treatment. Overall, HMDS surface modification effectively improves the compatibility of pigment-rich agro-waste fillers with PDMS and supports their use as functional fillers and natural colorant sources in silicone-based composites, providing a value-added route for the utilization of agro-industrial by-products.

## 1. Introduction

The generation of agro-industrial waste represents a major environmental and economic challenge worldwide. According to the Food and Agriculture Organization (FAO), approximately 1.3 billion tons of food are lost or wasted annually, accounting for nearly one-third of global food production [1,2]. In response, the European Green Deal and circular economy strategies encourage the reduction in food waste and the valorization of agri-food by-products through their incorporation into value-added materials and industrial applications [3]. The utilization of agricultural residues as renewable resources supports sustainable material development while reducing dependence on synthetic additives and non-renewable raw materials [4].

Agro-industrial by-products have been valorized through various approaches, including their use as animal feed, compost and organic fertilizers; in bioenergy production; and in the extraction of valuable bioactive compounds such as dietary fibers, antioxidants, and natural pigments [5]. While these strategies contribute to waste reduction and resource recovery, many require additional processing or specialized technologies. In comparison, the direct incorporation of these by-products as fillers in polymer composites offers a simple, cost-effective, and sustainable valorization route. Besides reducing agricultural waste, such fillers can partially replace conventional inorganic fillers while introducing functional properties, including natural coloration and reinforcement.

Fruit- and vegetable-processing by-products are rich in natural pigments, fibers, and other plant-derived constituents, making them attractive candidates for functional fillers and natural colorants in polymer systems [4,6]. Pigment-rich residues obtained from beetroot, raspberry, sea buckthorn, grape skins, and similar agro-industrial sources have gained increasing attention due to their abundance, low cost, and potential use as functional fillers and natural colorants in polymer composites [7,8]. Integrating agro-industrial by-products into polymeric materials adheres to circular economy principles by promoting waste valorization and resource efficiency while potentially enhancing material properties [9,10]. These materials can serve as functional fillers in polymer composites, providing both esthetic and performance-related benefits [11]. Natural pigments have been increasingly explored in textile, plastic, pharmaceutical, food-packaging, nanocomposite, and polymer applications as alternatives to synthetic colorants [11,12,13,14].

Among various polymer systems, polydimethylsiloxane (PDMS) has attracted significant attention for incorporating pigment-rich by-product fillers, primarily due to its transparency and capacity to preserve and enhance coloration [15]. In addition to its optical transparency, PDMS is generally regarded as chemically inert, non-flammable, and highly stable under a wide range of environmental conditions [16]. Owing to its exceptional physical and chemical properties, PDMS is widely used across industries such as automotive, electronics, healthcare, and personal care. Its high thermal stability, chemical resistance, and flexibility make it a preferred material for high-performance applications [17]. Its transparent nature also makes it a suitable matrix for incorporating naturally derived pigments and colored fillers.

In addition to serving as a carrier for naturally derived pigments, PDMS provides a versatile platform for evaluating the influence of surface-modified fillers on interfacial interactions, hydrophobicity, and mechanical performance. The objective of incorporating agro-industrial by-products into PDMS is not to impart biodegradability to the silicone matrix, but rather to utilize these renewable resources as functional fillers and colorant sources in high-value silicone-based materials. This approach enables the direct utilization of minimally processed agro-industrial residues in durable silicone composites, providing both functional performance and value-added utilization without the need for extensive extraction or purification processes.

Several studies have investigated the incorporation of natural fillers, including cellulose, lignocellulosic fibers, agricultural residues, and plant-derived particles, into PDMS matrices to improve mechanical performance, tailor surface properties, and enhance the sustainability of silicone-based materials [18]. However, the inherently hydrophilic nature of these fillers often results in poor compatibility with the hydrophobic PDMS matrix, leading to filler agglomeration and weak interfacial interactions. Consequently, surface modification using silane coupling agents, particularly HMDS, has emerged as an effective strategy to reduce filler surface polarity, improve filler dispersion, and enhance compatibility with silicone matrices.

To overcome the compatibility challenges associated with hydrophilic plant-based fillers in PDMS, surface modification techniques, particularly silanization, are widely employed to improve filler–matrix interactions and dispersion within hydrophobic polymer matrices [8,19,20,21]. Hexamethyldisilazane (HMDS) is an effective silane agent that reacts with hydroxyl groups on lignocellulosic surfaces, substituting them with hydrophobic Si(CH_3_)_3_ groups, thereby reducing polarity and strengthening interfacial interactions [22]. Silylation with HMDS changes the surface chemical composition of natural fillers (powder), reducing their polarity and enhancing filler–matrix interactions [19,22]. It has been shown that silylating natural fillers enhances their dispersion within polymer matrices and may improve interfacial adhesion and composite performance. Therefore, applying similar surface-functionalization approaches to pigment-rich plant powders may improve their dispersion and compatibility within hydrophobic polymer matrices [23]. Nevertheless, limited research has examined the HMDS modification of pigment-rich agro-industrial by-products intended for use as functional fillers and natural colorants in silicone-based matrices such as PDMS.

Although HMDS surface modification has been widely investigated for conventional lignocellulosic fillers such as cellulose fibers, wood flour, and agricultural residues, studies focusing on pigment-rich agro-industrial by-products remain scarce. Unlike conventional natural fillers, pigment-rich by-products offer a dual functionality by simultaneously acting as reinforcing agents and natural colorants. Furthermore, comparative studies evaluating multiple pigment-rich agro-industrial wastes under identical HMDS modification and PDMS processing conditions have not been reported. Therefore, the present work addresses this research gap by comparatively investigating four pigment-rich agro-industrial by-products following vapor-phase HMDS treatment and evaluating their effectiveness as multifunctional fillers in PDMS composites.

In this study, four pigment-rich agro-industrial by-products (beetroot, raspberry, sea buckthorn, and shadbush) were surface-modified using vapor-phase HMDS and incorporated into PDMS composites. Unlike previous studies focusing primarily on conventional lignocellulosic fillers, this work provides a comparative evaluation of pigment-rich wastes as multifunctional fillers capable of simultaneously reinforcing the polymer matrix while imparting natural coloration. The effects of HMDS treatment on filler morphology, surface chemistry, hydrophobicity, dispersion behavior, and puncture resistance were systematically investigated to establish the relationship between filler characteristics and composite performance.

## 2. Results and Discussion

### 2.1. Preparation of Composites

**Figure 1:**
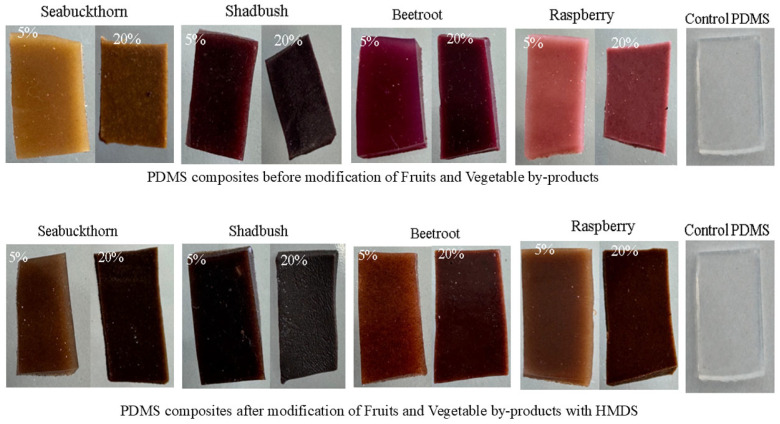
Digital photographs of PDMS composites containing sea buckthorn, shadbush, beetroot, and raspberry by-product fillers at 5 wt.% and 20 wt.% loadings before and after HMDS surface modification. Neat PDMS is shown as the control sample.

The visual appearance of the prepared composites is shown in Figure 1. Neat PDMS appeared translucent, whereas the incorporation of pigment-rich fruit and vegetable by-product fillers imparted distinct colors to the composites. The composite color became progressively darker with increasing filler loading, particularly at 20 wt.%, due to the higher concentration of natural pigments within the PDMS matrix. This effect is primarily attributed to the higher concentration of natural pigments at high filler loadings of 20 wt.%, which increase light absorption and reduce light transmission through the material [24]. In addition, increased filler content enhances light scattering due to the refractive index mismatch between the polymer matrix and the dispersed particles, resulting in reduced translucency and a darker visual appearance [25]. Furthermore, at higher filler loadings, partial particle agglomeration and reduced dispersion uniformity can occur, which further contributes to non-uniform light distribution and increased color intensity. Similar trends have been reported in polymer composite systems, where increasing pigment or filler concentration leads to decreased translucency and more saturated, or darker, shades due to enhanced light absorption and scattering [26]. HMDS treatment did not eliminate the natural coloration of the fillers, although slight variations in color intensity were observed depending on filler type and loading.

### 2.2. Morphological Analysis of Raw and Treated Fruits and Vegetables By-Product Powder

**Figure 2:**
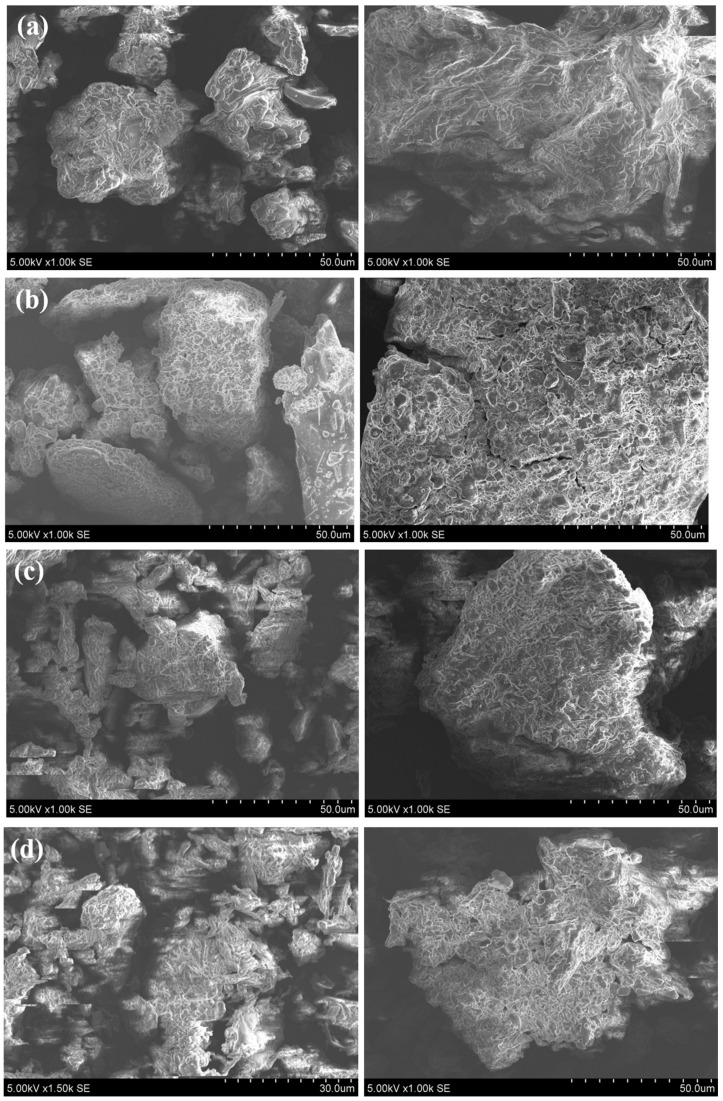
Scanning electron micrographs (SEM) of untreated and HMDS-treated pigment-rich fruit and vegetable by-product powders. The images on the left correspond to HMDS-treated powders, while the images on the right show untreated powders: (**a**) beetroot, (**b**) sea buckthorn, (**c**) shadbush, and (**d**) raspberry powders.

The surface morphology of untreated and surface-modified fruits and vegetable by-product powders, such as beetroot, shadbush, raspberry, and sea buckthorn, was investigated using scanning electron microscopy (SEM), as shown in Figure 2. Untreated powders typically exhibited agglomerated fragments and irregularly shaped particles with comparatively compact surfaces. These morphological characteristics refer to lignocellulosic fruit biomass, with surfaces partially coated by waxes, pectin, hemicellulose, and lignin. Berry pomace powders are known to contain significant amounts of cellulose, hemicellulose, lignin, and pectin originating from fruit skins and seeds, which contribute to their heterogeneous microstructure [27,28]. In lignocellulosic biomass materials, the presence of these structural polymers often leads to irregular particle morphology and aggregated clusters observed in SEM images [28].

Such surface layers often limit surface roughness and reduce interfacial compatibility with polar polymer matrices because lignocellulosic materials contain surface impurities and hydrophilic functional groups that hinder effective wetting by non-polar polymers [20,29]. All powders showed noticeable changes in particle morphology following surface modification. Compared with their untreated counterparts, the treated particles typically exhibit higher surface roughness, fibrillation, and noticeable microscale abnormalities. This change is due to a modification in the outer layer, with some surface material removed, exposing the inner cellulose fibers and making the surface more irregular. Similar observations were reported for silane-treated lignocellulosic fibers, where surface cleaning and microfibril exposure led to rougher surfaces and improved interfacial interactions with polymer matrices [30]. While all powders showed a general tendency to increase roughness after modification, there were significant differences in the morphological changes between the samples.

With treatment, the raspberry (Figure 2d) and shadbush (Figure 2c) powders exhibit more pronounced surface fibrillation and asymmetrical microstructures, suggesting a more severe breakdown of the external cell wall structure. The high dietary fiber and seed-derived lignocellulosic content in berry pomace tends to form heterogeneous, fibrillar structures after processing. Due to the presence of cellulose-rich seed and skin fractions, studies on berry pomace powders have reported comparable irregular, fiber-rich morphologies [31]. On the other hand, after treatment, the beetroot powder (Figure 2a) shows comparatively more compact particles with considerable surface roughening. This pattern could be related to beetroot’s higher pectin and parenchymal tissue composition, which creates more continuous cellular matrices, while the by-products of berry are less uniform and have more fibrous structures [32]. Overall, the SEM results indicate that the surface modification method successfully alters the morphology of the investigated powders by changing their surface irregularity and microscale roughness. In PDMS polymer matrices, these structural alterations improve filler dispersion and encourage mechanical interlocking. Filler–matrix interactions and interfacial bonding in composite systems are known to improve with increased surface roughness and greater exposure of cellulose microfibrils.

Following treatment, the sea buckthorn Figure 2b powder exhibits microscale pores and fissures, resulting in a heterogeneous, porous surface morphology. Similar microstructural changes have been reported for sea buckthorn pomace, where drying and mechanical processing can cause partial collapse of the cellular structure and the formation of pores and cracks in the lignocellulosic matrix [33,34]. These observations further support the effectiveness of HMDS treatment in modifying the surface morphology of agro-industrial by-product powders and are consistent with the trends observed for the other investigated materials.

### 2.3. FTIR Spectra of Untreated and HMDS-Treated Fruits and Vegetable By-Products

**Figure 3:**
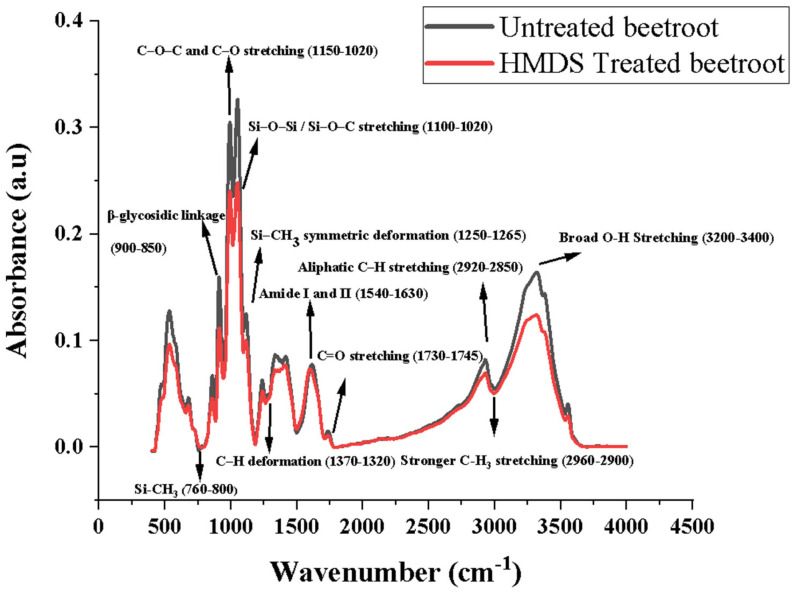
Absorbance spectra (4000–600 cm^−1^, 4 cm^−1^ resolution, Nicolet iN10 microscope) of untreated and HMDS-treated beetroot powders. Characteristic absorption bands include O–H stretching (3200–3500 cm^−1^), Si–CH_3_ vibrations (840–1250 cm^−1^), and Si–O–Si stretching (1000–1100 cm^−1^). The reduced O–H band intensity and the appearance/enhancement of Si–CH_3_ and Si–O–Si bands confirm successful HMDS-induced silylation.

**Figure 4:**
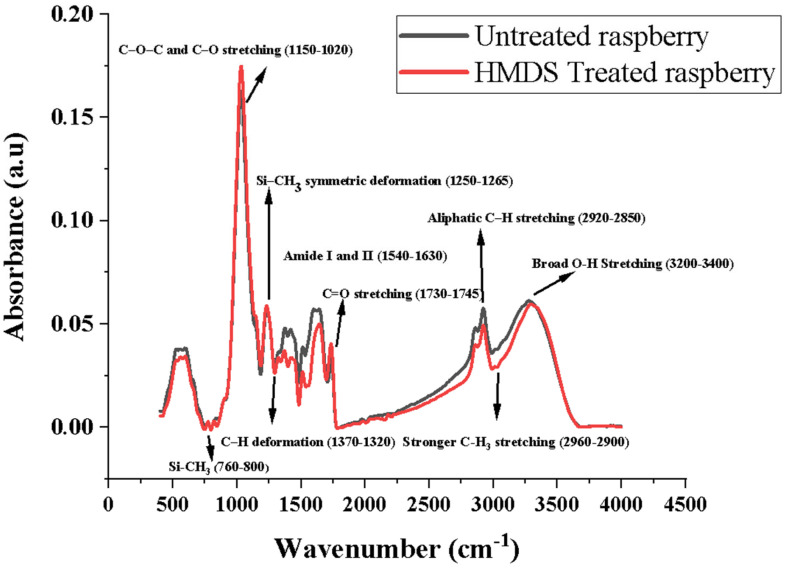
Absorbance spectra (4000–600 cm^−1^, 4 cm^−1^ resolution, Nicolet iN10 microscope) of untreated and HMDS-treated raspberry powders. Characteristic absorption bands include O–H stretching (3200–3500 cm^−1^), Si–CH_3_ vibrations (840–1250 cm^−1^), and Si–O–Si stretching (1000–1100 cm^−1^). The reduced O–H band intensity and the appearance/enhancement of Si–CH_3_ and Si–O–Si bands confirm successful HMDS-induced silylation.

**Figure 5:**
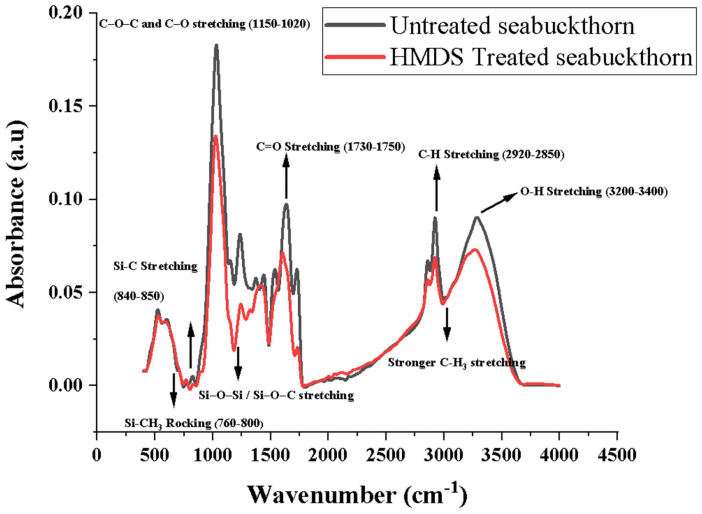
Absorbance spectra (4000–600 cm^−1^, 4 cm^−1^ resolution, Nicolet iN10 microscope) of untreated and HMDS-treated sea buckthorn powders. Characteristic absorption bands include O–H stretching (3200–3500 cm^−1^), Si–CH_3_ vibrations (840–1250 cm^−1^), and Si–O–Si stretching (1000–1100 cm^−1^). The reduced O–H band intensity and the appearance/enhancement of Si–CH_3_ and Si–O–Si bands confirm successful HMDS-induced silylation.

**Figure 6:**
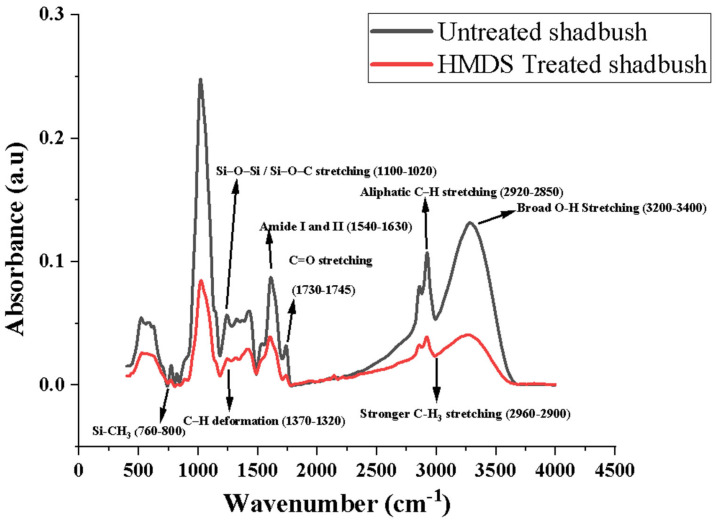
Absorbance spectra (4000–600 cm^−1^, 4 cm^−1^ resolution, Nicolet iN10 microscope) of untreated and HMDS-treated shadbush powders. Characteristic absorption bands include O–H stretching (3200–3500 cm^−1^), Si–CH_3_ vibrations (840–1250 cm^−1^), and Si–O–Si stretching (1000–1100 cm^−1^). The reduced O–H band intensity and the appearance/enhancement of Si–CH_3_ and Si–O–Si bands confirm successful HMDS-induced silylation.

The FTIR (Fourier-transform infrared) spectra of beetroot, raspberry, sea buckthorn, and shadbush are presented in Figure 3, Figure 4, Figure 5 and Figure 6, indicating the effect of HMDS surface modification. Successful HMDS-induced surface modification was confirmed by distinct changes in the FTIR spectra. Raspberry and sea buckthorn samples, in Figure 4 and Figure 5, exhibited peak broadening and reduced intensity in the O–H region (~3300 cm^−1^), and this reduction is attributed to –OH peak intensity (3200–3550 cm^−1^) due to silylation: –OH replaced by –O–Si(CH_3_)_3_. A slight increase around 850–950 cm^−1^ corresponds to Si–CH_3_ groups from HMDS. The reduction in hydroxyl group intensity indicates decreased surface polarity, which is expected to improve the affinity of the modified fillers toward the hydrophobic PDMS matrix [35].

In the beetroot samples in Figure 3, the Si–O–Si and hydrophilic groups (O–H and C=O) were reduced, indicating effective surface modification of the powder [36]. The FTIR spectra of shadbush samples in Figure 6 revealed a noticeable decrease in absorbance at 1700 cm^−1^ (C=O) and 3300–3400 cm^−1^ (O–H), along with the appearance of new peaks between 800 and 1000 cm^−1^, corresponding to the incorporation of Si–O and Si–C bonds. HMDS treatment replaces polar surface hydroxyl (–OH) groups with non-polar –Si(CH_3_)_3_ groups, thereby reducing surface energy and increasing hydrophobicity. This is confirmed by FTIR through the decrease in the O–H band and the appearance of Si–CH_3_-related peaks [37]. Since berry powders are rich in hydroxyl-containing compounds, a similar silylation effect is expected, supporting the effectiveness of HMDS treatment, as similar behavior has been reported for HMDS-modified silica and polysaccharide-based materials, where the removal of hydroxyl groups leads to improved hydrophobicity and compatibility with non-polar matrices [38]. Figure 3, Figure 4, Figure 5 and Figure 6 show that the O–H peak (3200–3600 cm^−1^) becomes weaker and the Si–CH_3_ peaks (~1250 and 840 cm^−1^) become stronger after HMDS treatment. The antisymmetric C–H stretching vibrations give rise to absorption bands at 2960 cm^−1^ and 2910 cm^−1^, corresponding to the C-H_3_ and C-H_2_ groups, respectively. All the HMDS-treated powders show Si–O–Si bands around ~1000–1100 cm^−1^, while the 1260 cm^−1^ band is reported for methyl groups attached to silicon [39]. The appearance of the Si–O–Si band is attributed to the silylation reaction between the surface hydroxyl (–OH) groups present on the lignocellulosic constituents of the by-product powders and HMDS. During this reaction, hydroxyl groups are replaced by trimethylsilyl (–Si(CH_3_)_3_) groups, forming Si–O–Si linkages while releasing ammonia (NH_3_) as a by-product. The formation of these linkages reduces the surface polarity of the powders and contributes to their improved compatibility with the hydrophobic PDMS matrix. The absorption observed at 700 cm^−1^ corresponds to C–C stretching vibrations [40]. The appearance of these silicon-containing functional groups is consistent with the increased hydrophobicity observed in the contact angle measurements following HMDS treatment [41].

These findings are consistent with previous studies on HMDS-modified lignocellulosic and silica-based materials, where replacement of surface hydroxyl groups with hydrophobic trimethylsilyl groups resulted in reduced surface polarity and enhanced compatibility with hydrophobic matrices [37,38]. The decrease in O–H band intensity together with the appearance of Si–CH_3_- and Si–O–Si-related bands confirms the successful silylation of the pigment-rich by-product powders.

Overall, all four pigment-rich by-product powders exhibited similar FTIR trends following HMDS treatment. A noticeable reduction in the intensity of the broad O–H stretching band (3200–3400 cm^−1^), together with the appearance or enhancement of characteristic Si–CH_3_ (760–800 and 1250–1265 cm^−1^) and Si–O–Si/Si–O–C (1020–1100 cm^−1^) bands, confirms successful HMDS-induced silylation. These spectral changes qualitatively indicate the replacement of surface hydroxyl groups by hydrophobic trimethylsilyl moieties, thereby reducing surface polarity, enhancing hydrophobicity, and improving the potential compatibility of the fillers with the PDMS matrix.

### 2.4. Color Analysis

**Figure 7:**
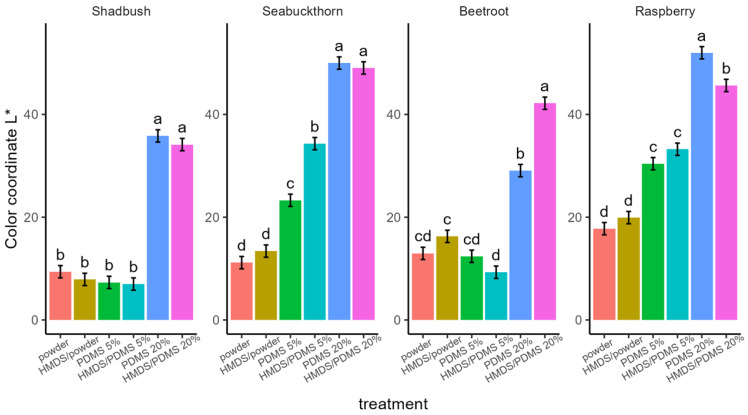
Colorimetric parameters (L*) of PDMS composites (inferred from text). CIE L* (lightness) values for raw powders, HMDS-treated powders, and PDMS composites at 5 wt.% and 20 wt.% loadings. Bars represent mean ± standard deviation (SD, *n* = 3). Different letters indicate significant differences (*p* < 0.05) according to Tukey’s honestly significant difference (HSD) test.

**Figure 8:**
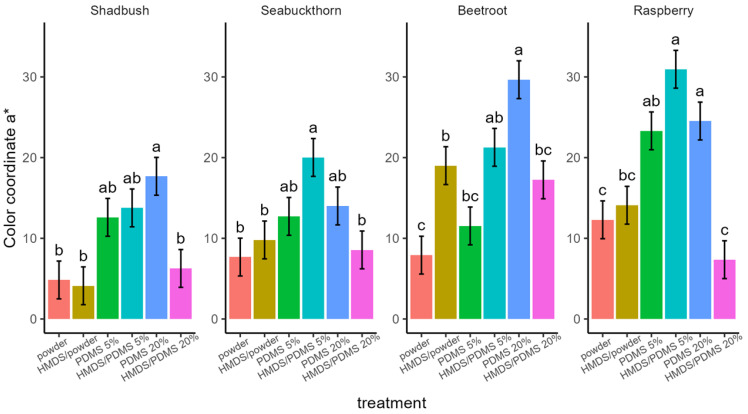
Colorimetric parameters (a*) of PDMS composites (inferred from text). CIE a* (red, green) values for raw powders, HMDS-treated powders, and PDMS composites at 5 wt.% and 20 wt.% loadings. Bars represent mean ± standard deviation (SD, *n* = 3). Different letters indicate significant differences (*p* < 0.05) according to Tukey’s honestly significant difference (HSD) test.

**Figure 9:**
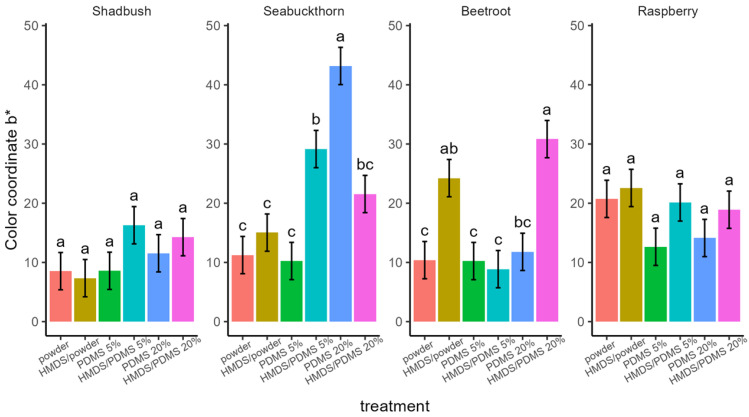
Colorimetric parameters (b*) of PDMS composites (inferred from text). CIE b* (yellow, blue) values for raw powders, HMDS-treated powders, and PDMS composites at 5 wt.% and 20 wt.% pigment rich filler loadings. Bars represent mean ± standard deviation (SD, *n* = 3). Different letters indicate significant differences (*p* < 0.05) according to Tukey’s honestly significant difference (HSD) test.

The chromatic coordinates L* (lightness), a* (red–green), and b* (yellow–blue) of raw and modified fruits and vegetable powders along with the PDMS composites are presented in Figure 7, Figure 8 and Figure 9. The incorporation of pigment-rich agro-waste fillers significantly influenced the color characteristics of the composites, depending on filler type, surface treatment, and loading level. A general trend of darker shades with increasing filler loading (20 wt.% vs. 5 wt.%) was observed across all samples. This behavior can be attributed to the higher concentration of natural pigments at elevated wt.%, which increases light absorption and reduces light transmission through the composite. In addition, increased filler content enhances light scattering due to the refractive index mismatch between the PDMS matrix and dispersed particles, resulting in reduced translucency and a darker visual appearance. Similar trends have been reported in polymer composites containing natural pigments, where increased pigment concentration leads to reduced lightness and greater color saturation [25]. The L* values showed variation depending on both filler type and HMDS treatment. In general, composites prepared with HMDS-treated fillers exhibited lower L* values (darker appearance) compared to untreated systems at the same loading, particularly at 20 wt.%. This suggests improved pigment–matrix compatibility and more efficient pigment dispersion, resulting in greater light absorption within the composite. However, at lower loading, 5 wt.% more uniform and slightly lighter shades were observed, likely due to better dispersion and reduced particle agglomeration. The increase in lightness upon incorporation into the PDMS matrix, particularly with HMDS Figure 7, suggests improved pigment dispersion and the formation of more light-reflective composite surfaces. It was also shown, in Ibáñez-García et. al., that the biopolymer matrix was affected by the increase in color masterbatch concentration [8]. It is clear from the color variation that the tendency to increase is evident in treated powder and composites made with it. In Figure 7, the beetroot HMDS treatment shows no significant difference from the raw powder treatment (*p* = 0.387, not significant), as beetroot already exhibits high color intensity. Furthermore, 20 wt.% PDMS treatment significantly increases the L* lightness index as compared to the raw powder treatment (*p* < 0.001). The 20 wt.% HMDS/PDMS treatment revealed a noticeable increase in lightness L* intensity (*p* < 0.001).

The increase in the a* coordinate presented in Figure 8 indicates stronger red coloration due to the presence of natural pigments such as anthocyanins and carotenoids. Similar trends have been reported in polymer systems containing plant pigments, where incorporation of natural pigments significantly alters the CIE L*, a*, and b* color coordinates and increases redness depending on pigment concentration and dispersion within the matrix [42]. Better pigment dispersion and visibility may be possible with 5 wt.% PDMS, whereas 20 wt.% PDMS occasionally obscures color with the matrix’s bulk. The reported effects of pumice, scoria, and MWCNT fillers in PDMS are that mineral and carbon-based additives impart distinct shades and improve filler visibility [24].

The increase in the b* coordinate shown in Figure 9 observed for sea buckthorn containing composites indicates enhanced yellowness associated with carotenoid pigments, which are abundant in sea buckthorns and responsible for yellow-orange coloration [43]. Incorporation of natural pigments into polymer matrices has been reported to significantly modify the CIE L*, a*, and b* color coordinates depending on pigment dispersion and concentration [8]. In addition, hydrophobic environments may improve carotenoid stability and visibility, which may explain the higher b* values observed in HMDS-treated systems due to improved pigment–matrix interactions [44]. According to earlier research on PDMS composites, in which the final optical appearance is determined by the filler’s intrinsic color and dispersion quality, the pigment-rich byproducts in this study produced distinct coloration patterns [45]. By improving filler–matrix compatibility and pigment stability, HMDS surface modification increased L*, a*, and b* values. The current investigation by hydrophobic silane functionalization maintained pigment integrity and decreased light scattering at the filler–matrix interface [46].

### 2.5. EDS Analysis of HMDS-Treated Fruit and Vegetable By-Product Powders

**Figure 10:**
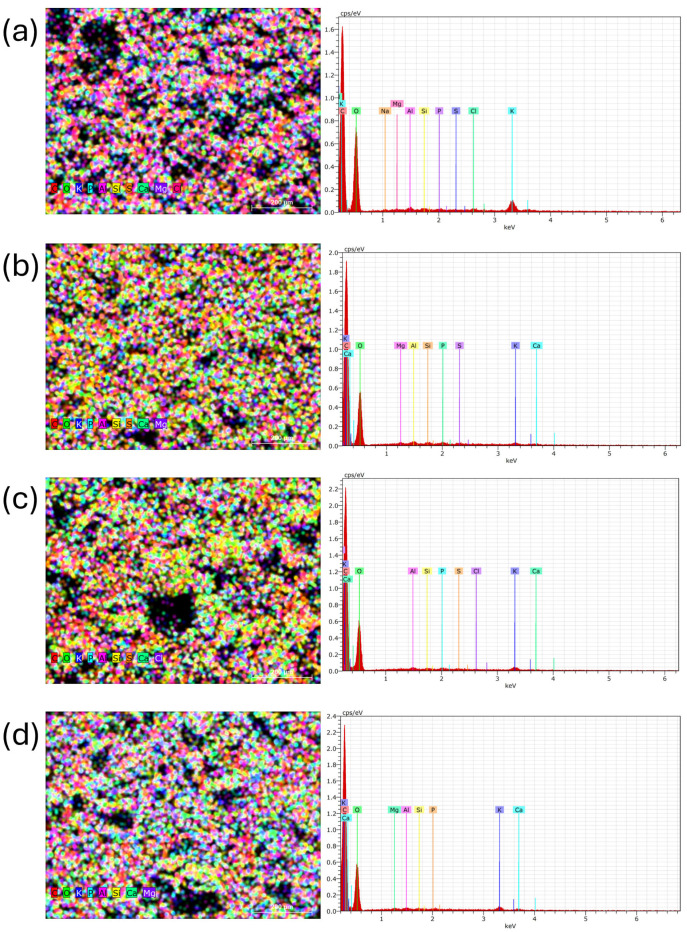
EDS elemental mapping and EDS spectra of HMDS-treated powders: (**a**) beetroot, (**b**) raspberry, (**c**) sea buckthorn, and (**d**) shadbush. The presence of silicon (~1.7 keV) confirms HMDS-induced surface modification.

EDS elemental mapping and spectra of the HMDS-treated fruit and vegetable by-product powders are presented in Figure 10. Carbon and oxygen were the predominant elements detected in all samples, with characteristic peaks observed at approximately 0.28 and 0.52 keV, respectively, reflecting the organic composition of the plant-derived materials [47]. In addition, silicon was detected in beetroot (0.26 wt.%), sea buckthorn (0.25 wt.%), raspberry (0.16 wt.%), and shadbush (0.11 wt.%). Detailed elemental composition data are provided in Tables S1–S4. The EDS spectra exhibited a characteristic silicon peak at approximately 1.7 keV, corresponding to the Si Kα emission line reported in previous studies [48]. The presence of silicon on the powder surfaces is consistent with the incorporation of silicon-containing groups following HMDS treatment. Furthermore, elemental mapping showed the distribution of silicon across the treated powder surfaces, which is consistent with successful HMDS surface modification. These findings, together with the FTIR results showing the appearance of silicon-containing functional groups after treatment, provide complementary qualitative evidence supporting HMDS-induced surface silylation. Although comparative EDS analysis of untreated powders was not performed, the combined EDS and FTIR results consistently support successful HMDS surface modification of the pigment-rich by-product powders.

### 2.6. Water Contact Angle Characterization

**Figure 11:**
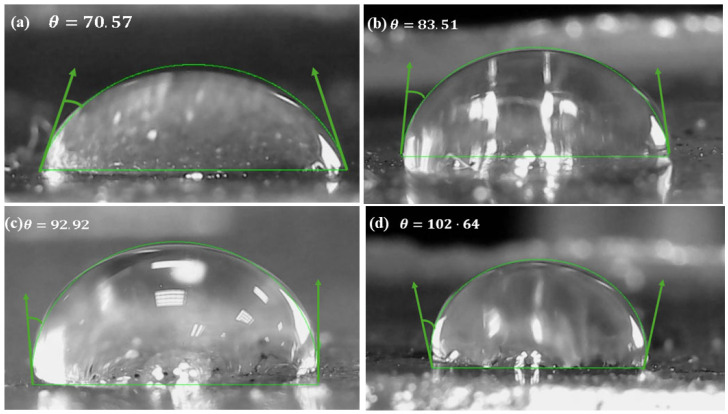
Sessile drop images and contact angle measurements (°) for PDMS composites with shadbush by-product fillers at 5 wt.% and 20 wt.% loadings with raw (**a**,**b**) upper images and HMDS-treated (**c**,**d**) lower images. Angles measured via ImageJ; mean values shown.

**Figure 12:**
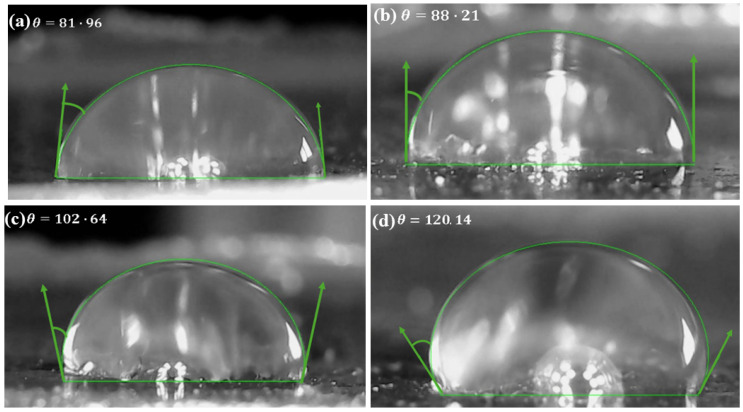
Sessile drop images and contact angle measurements (°) for PDMS composites with beetroot by-product fillers at 5 wt.% and 20 wt.% loadings with raw (**a**,**b**) upper images and HMDS-treated (**c**,**d**) lower images. Angles measured via ImageJ; mean values shown.

The wettability of the composite’s surfaces was evaluated by measuring contact angles with ImageJ version 1.54g. In general, surfaces exhibiting water contact angles of approximately 90° or greater are considered hydrophobic, whereas those with contact angles below 90° are regarded as hydrophilic [49]. For comparison, the water contact angle of neat PDMS was also measured and is provided in the Supplementary Materials (Figure S1). The neat PDMS surface exhibited a contact angle of 91.08°, confirming the inherently hydrophobic nature of the silicone matrix. Therefore, changes in composite wettability can be attributed to the presence of the agro-industrial by-product fillers and the effects of HMDS surface modification. The contact angles of PDMS composites with and without HMDS-treated shadbush powder as fillers are presented in Figure 11. At a filler loading of 5 wt.%, the contact angle was 70.57°, which increased to 83.51° when the filler loading was raised to 20 wt.%. In contrast, HMDS-treated PDMS composites exhibited a further significant increase in contact angle, reaching 92.92° and 102.84° at 5 wt.% and 20 wt.% filler loadings, respectively. This increase is attributed to surface modification induced by HMDS treatment, which rendered the shadbush filler more hydrophobic. A similar trend was observed for beetroot fillers, as shown in Figure 12. The contact angles of PDMS composites without HMDS treatment were 81.96° and 88.21° at 5 wt.% and 20 wt.% filler loadings, respectively. However, upon incorporation of HMDS, the contact angles increased markedly to 102.64° and 120.14°, indicating the formation of a highly hydrophobic surface.

**Figure 13:**
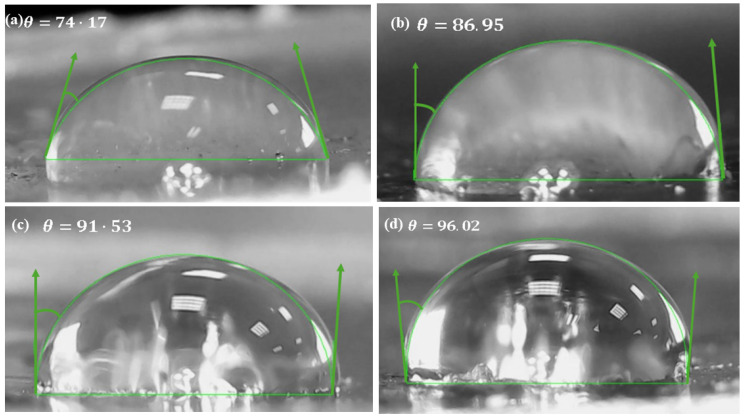
Sessile drop images and contact angle measurements (°) for PDMS composites with sea buckthorn by-product fillers at 5 wt.% and 20 wt.% loadings with raw (**a**,**b**) upper images and HMDS-treated (**c**,**d**) lower images. Angles measured via ImageJ; mean values shown.

Sea buckthorn-filled composites exhibited a similar increase in hydrophobicity following HMDS treatment, as shown in Figure 13. The contact angles of untreated composites increased from 74.17° at 5 wt.% filler loading to 86.95° at 20 wt.% filler loading. After HMDS treatment, the contact angles further increased to 91.53° and 96.02° for 5 wt.% and 20 wt.% filler loadings, respectively. These results indicate that HMDS treatment effectively reduced the surface polarity of sea buckthorn fillers, resulting in enhanced hydrophobicity and improved compatibility with the PDMS matrix.

**Figure 14:**
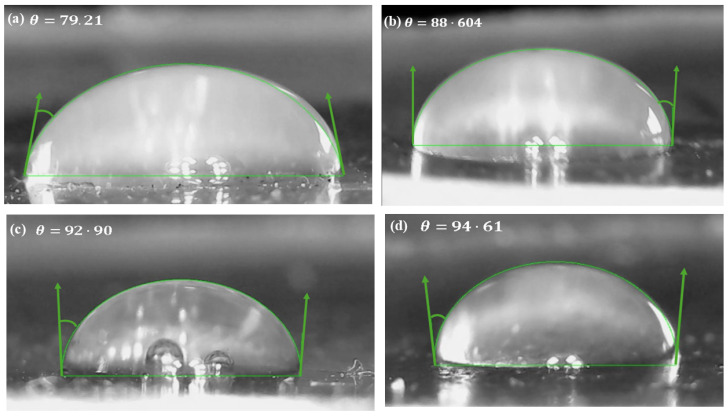
Sessile drop images and contact angle measurements (°) for PDMS composites with raspberry by-product fillers at 5 wt.% and 20 wt.% loadings with raw (**a**,**b**) upper images and HMDS-treated (**c**,**d**) lower images. Angles measured via ImageJ; mean values shown.

The contact angles of raspberry-filled composites shown in Figure 14 were 79.21° and 88.60° at 5 wt.% and 20 wt.% filler loadings, respectively, and increased to 92.90° and 94.61° following HMDS treatment. The increase in contact angle after HMDS treatment is attributed to surface silanization, which replaces hydroxyl groups on the filler surface with hydrophobic trimethylsilyl groups (–Si(CH_3_)_3_), thereby reducing surface polarity and improving compatibility with the hydrophobic PDMS matrix [50,51]. Although PDMS is intrinsically hydrophobic, the measured contact angles of the untreated composites remained below 90° because the hydrophilic filler particles were exposed at or near the composite surface. Consequently, the wettability of the composites was influenced not only by the PDMS matrix but also by the surface distribution and chemistry of the filler particles. This suggests that the exposed filler particles partially governed the surface properties of the composites despite the high PDMS content. In addition, higher filler loadings contributed to increased surface hydrophobicity, likely due to changes in surface roughness and filler distribution within the composite. These findings are consistent with previous studies reporting enhanced hydrophobicity of natural fillers following HMDS surface modification [52,53]. The observed increase in the contact angle further supports the FTIR results, which confirmed the introduction of hydrophobic silicon-containing groups on the filler surfaces. The substantial increase in the contact angle after HMDS treatment indicates that surface chemistry, rather than PDMS content alone, played a major role in determining composite wettability. These findings are consistent with Avrămescu et al., who reported that hydrophobic surfaces possessing low surface energy promote the formation of nearly spherical water droplets with contact angles of approximately 90° or greater [54]. Across all filler types, contact angles generally increased as filler loading increased from 5 wt.% to 20 wt.%, which may be associated with changes in surface roughness and filler distribution within the composite. Following HMDS treatment, the filler surfaces became less polar due to the introduction of trimethylsilyl groups, resulting in increased hydrophobicity and improved compatibility with the PDMS matrix. These observations are consistent with the findings of Shiroud Heidari et al., who reported that cellulose microfibers silylated with HMDS via vapor-phase treatment exhibited enhanced hydrophobicity, with contact angles increasing from approximately 95° to 117° ± 2°. Similarly, the HMDS-treated pigment-rich fillers investigated in the present study produced composite contact angles ranging from approximately 91° to 120°, depending on the filler type and loading. The slight differences between the two studies may be attributed to variations in filler composition, particle morphology, surface chemistry, HMDS treatment conditions, and the influence of the PDMS matrix on the overall surface wettability of the composites [55].

Improved filler hydrophobicity may promote better interfacial interactions and greater compatibility with the silicone matrix, which may also contribute to improved filler distribution within the composite.

### 2.7. Morphological Properties of Composites

**Figure 15:**
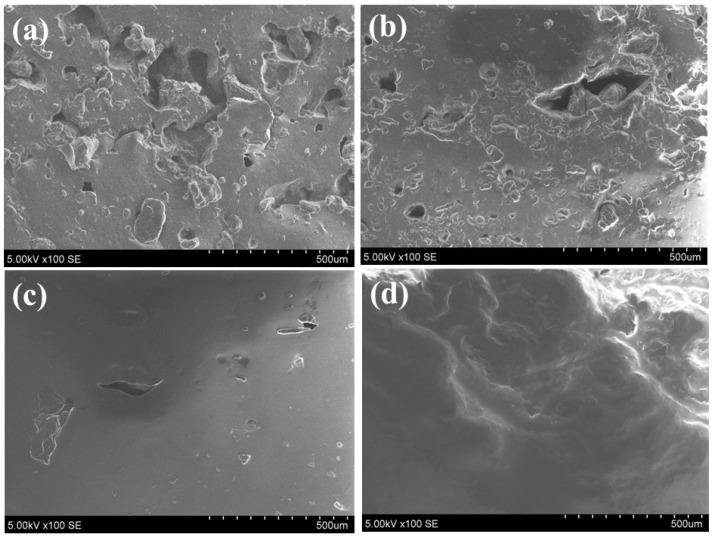
SEMs of raspberry-filled PDMS composites: (**a**) untreated filler (5 wt.%), (**b**) untreated filler (20 wt.%), (**c**) HMDS-treated filler (5 wt.%), and (**d**) HMDS-treated filler (20 wt.%). Scale bars: 500 µm.

**Figure 16:**
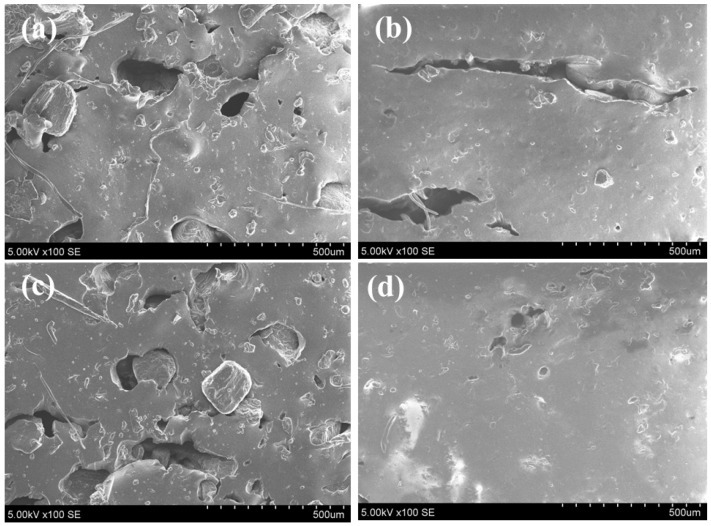
SEMs of sea buckthorn-filled PDMS composites: (**a**) untreated filler (5 wt.%), (**b**) untreated filler (20 wt.%), (**c**) HMDS-treated filler (5 wt.%), and (**d**) HMDS-treated filler (20 wt.%). Scale bars: 500 µm.

**Figure 17:**
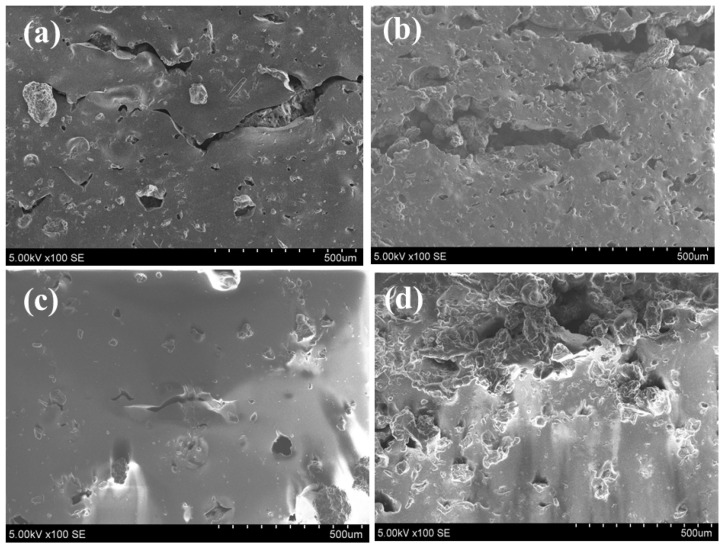
SEMs of shadbush-filled PDMS composites: (**a**) untreated filler (5 wt.%), (**b**) untreated filler (20 wt.%), (**c**) HMDS-treated filler (5 wt.%), and (**d**) HMDS-treated filler (20 wt.%). Scale bars: 500 µm.

**Figure 18:**
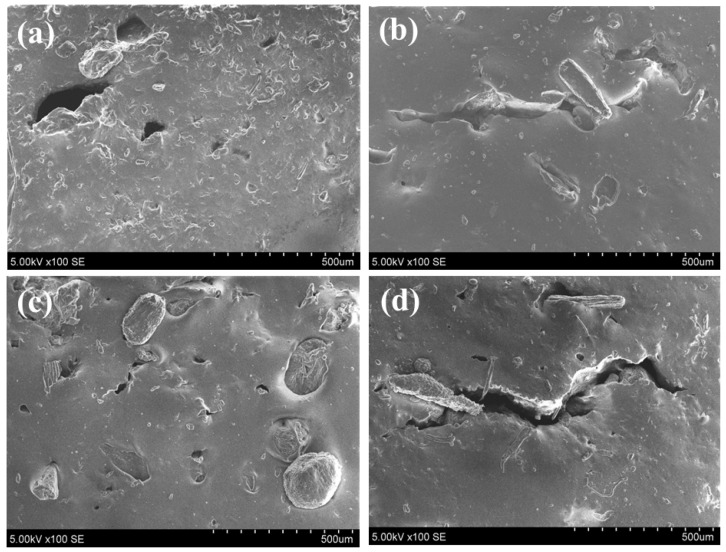
SEMs of beetroot-filled PDMS composites: (**a**) untreated filler (5 wt.%), (**b**) untreated filler (20 wt.%), (**c**) HMDS-treated filler (5 wt.%), and (**d**) HMDS-treated filler (20 wt.%). Scale bars: 500 µm.

SEM (scanning electron microscopy) was used to evaluate the surface morphology and provide qualitative information regarding filler distribution and the filler–matrix interface of shadbush, sea buckthorn, beetroot, and raspberry powders in PDMS composites with filler loadings of 5 wt.% and 20 wt.%, with and without HMDS modification, Figure 15, Figure 16, Figure 17 and Figure 18. These microstructures illustrate how filler dispersion, surface roughness, and interfacial compatibility are influenced by surface modification and polymer content. PDMS composites containing untreated powders, particularly at higher filler loadings, exhibited heterogeneous fracture surfaces characterized by filler agglomeration, interfacial gaps or pull-outs, and void formation across all four by-products (shadbush, sea buckthorn, beetroot, and raspberry), as shown in Figure 15, Figure 16, Figure 17 and Figure 18. These characteristics, which lead to poor wetting and weak interfacial interactions, are commonly observed when hydrophilic lignocellulosic particles are dispersed within hydrophobic PDMS matrices. Similar interfacial morphologies have frequently been reported for natural fibers incorporated into hydrophobic polymer matrices prior to silane surface treatment [20,56]. Modification of fruit and vegetable by-products with HMDS appeared to produce smoother and more continuous matrix coverage of the particles with fewer interfacial gaps and less evidence of particle pull-out, most notably in the 5 wt.% PDMS with HMDS-treated filler composites. This observation aligns with the reported mechanism in which silanes, featuring trimethylsilyl (-Si(CH_3_)_3_) groups, form covalent Si–O–Si bonds with surface –OH groups, thereby reducing surface energy and enhancing PDMS wetting and adhesion [57]. In a recent study, halloysite nanotubes (HNTs) were surface-modified with HMDS and trimethylchlorosilane (TMCS) to enhance hydrophobicity. The surface morphology was observed by SEM, and it was found that epoxy coatings containing pristine HNTs (10 wt.% and 20 wt.%) exhibited aggregation. In contrast, no aggregation was observed in epoxy coatings containing modified HNTs (m-HNTs). These results reported in the literature indicate that TMCS and HMDS treatments improved filler dispersion [58]. In the present study, SEM observations showed similar morphological trends; however, quantitative particle size distribution and zeta potential analyses were not performed.

In another study, superhydrophobic coatings were produced by combining SiO_2_ nanoparticles with silane modifiers and inorganic nanoparticles such as TiO_2_, generating hierarchical micro- and nanoscale roughness that significantly enhanced surface hydrophobicity and self-cleaning performance [22,59]. Cellulose microfibers treated with HMDS or silane in PDMS, and silane-modified inorganic fillers, have been shown to exhibit similar morphological improvements (better dispersion, reduced gaps at the particle–polymer interface). Surface treatment with silane was also performed to examine morphological trends in fiber/PDMS composites, thereby improving surface smoothness and increasing filler–rubber interaction [60]. HMDS reacts with hydroxyl-terminated silicon dioxide surfaces to form a hydrophobic (water-repellent) trimethylsilanol (TMSiOH) layer that improves photoresist adhesion [61].

Silane-based surface modification, including hexamethyldisilazane (HMDS) treatment, enhances filler hydrophobicity and compatibility with hydrophobic polymer matrices such as Polydimethylsiloxane by replacing surface hydroxyl (–OH) groups with low-surface-energy Si–CH_3_ groups and forming Si–O–Si linkages. This modification reduces surface polarity and improves interfacial adhesion, thereby enhancing dispersion and composite performance [21]. Recent studies have reported that silane-treated fillers exhibit increased water contact angles and improved interfacial bonding. In the present study, SEM observations are consistent with improved compatibility following HMDS treatment [55,62].

### 2.8. Mechanical Behavior Under Puncture Loading

Table 1 presents the mechanical properties of PDMS composites incorporating raw and surface-modified (HMDS-treated) fruit and vegetable by-product fillers. A texture analyzer was used to assess the mechanical behavior of PDMS composites reinforced with powders of untreated and HMDS-treated fruit and vegetable by-products. Puncture force, elasticity, and adhesiveness parameters represent the composite materials’ resistance to deformation. The filler type, concentration, and surface treatment had significant effects on the puncturing force values. The influence of HMDS treatment on mechanical performance was filler-dependent. Raspberry-filled composites exhibited improved puncture resistance and elasticity following HMDS treatment at low filler loading, whereas beetroot and shadbush composites showed reduced puncture resistance after treatment. The 5 wt.% PDMS-HMDS sample exhibited the highest puncture force, 252.78 N·cm^−2^, and elasticity, 19.22 N·cm^−2^·s^−1^, indicating enhanced filler–matrix interaction after surface modification. The improved performance of HMDS-treated raspberry filler may be associated with improved filler–matrix compatibility following HMDS treatment, as suggested by the SEM observations. However, increasing the filler loading to 20 wt.% reduced the puncture force to 133.22–135.49 N·cm^−2^, likely due to particle agglomeration and reduced matrix continuity. Beetroot composites exhibited relatively high puncture force values, with the 5 wt.% untreated composite reaching 252.99 N·cm^−2^. However, HMDS treatment reduced puncture resistance to 212.12 N·cm^−2^, indicating that surface modification did not enhance mechanical performance for this filler type.

Elasticity values remained relatively high: ~13–18 N·cm^−2^·s^−1^ for both treated and untreated samples. This behavior may be related to the pectin-rich parenchymal tissue structure of beetroot, which forms compact particles capable of reinforcing the PDMS matrix. Sea buckthorn-filled composites showed moderate mechanical properties, with puncture force values around 245 N·cm^−2^ for 5 wt.% PDMS and approximately 131 N·cm^−2^ for 20 wt.% PDMS composites. HMDS treatment improved elasticity but did not significantly alter puncture resistance. Adhesiveness decreased after treatment, suggesting reduced surface polarity due to silylation. Shadbush composites exhibited the greatest reduction in mechanical performance following HMDS treatment. The puncture force decreased from 200.72 N·cm^−2^ in the untreated composite to 93.43–85.10 N·cm^−2^ after treatment, accompanied by a reduction in elasticity (7.28–9.02 N·cm^−2^·s^−1^), indicating reduced flexibility. These results indicate that HMDS treatment does not universally improve composite mechanical properties and that the effectiveness of surface modification depends on filler composition, morphology, and interfacial interactions with the PDMS matrix. The improved mechanical performance observed for raspberry-filled composites may be attributed to more effective HMDS-induced surface modification, resulting in better compatibility with the PDMS matrix. In contrast, beetroot and shadbush powders may differ in their chemical composition, particle morphology, and surface characteristics, which could limit the effectiveness of HMDS treatment and contribute to the observed reduction in puncture resistance. These explanations are proposed based on the observed morphological differences and should be further verified through additional physicochemical characterization. All composites exhibited negative adhesiveness values, which is typical for silicone-based materials due to their viscoelastic recovery after deformation. Although HMDS treatment generally reduced adhesiveness values for raspberry and sea buckthorn composites, these changes were not statistically significant. This suggests that HMDS treatment primarily modified the surface characteristics of the fillers without substantially altering the inherent adhesive behavior of the PDMS matrix. Since PDMS possesses intrinsically low surface energy and elastomeric properties, the incorporation of untreated or HMDS-treated fillers at the investigated loadings had only a limited influence on the overall adhesiveness of the composites.

Experimental studies have demonstrated that silane treatment can significantly improve the tensile strength and modulus of natural fiber composites by enhancing filler–matrix bonding [63]. Similar improvements in mechanical resistance after silane modification of natural fillers have been widely reported for polymer composites. Silane coupling agents form chemical bridges between hydroxyl groups on lignocellulosic fibers and the polymer matrix, thereby enhancing interfacial adhesion and stress transfer within the composite [29]. Furthermore, the formation of Si–O–C and Si–O–Si linkages between treated fibers and polymer matrices has been shown to reduce interfacial defects and improve overall composite mechanical performance [64]. In the present study, however, the mechanical response varied among filler types, suggesting that the beneficial effects commonly associated with silane treatment may depend on the specific structural characteristics of the agro-industrial by-product used as filler.

However, at greater filler loadings of 20 wt.%, the puncture force generally decreased. This can be explained by less polymer chain mobility inside the matrix and particle agglomeration. Reduced matrix continuity and the possible formation of particle agglomerates at higher filler concentrations may have contributed to localized stress concentrations, thereby decreasing the mechanical integrity of the composites [16]. Findings from Ibáñez-García et al. indicate that tensile parameters, including Young’s modulus and tensile strength, did not change significantly when natural pigment-based color masterbatches were incorporated into PLA and PBS matrices at loadings of 2–6 wt.% compared with the untreated polymers [8]. The puncture test was selected to evaluate the localized deformation resistance of the PDMS composites under concentrated loading, which is relevant for flexible silicone materials subjected to indentation or puncture during handling and practical applications. Although conventional tensile tests provide complementary information such as tensile strength, Young’s modulus, and elongation at break, these measurements were beyond the scope of the present study. Future investigations should include these standardized mechanical tests to provide a more comprehensive mechanical characterization of the developed composites. Overall, the mechanical performance was strongly dependent on filler type and loading, highlighting the filler-specific effects of HMDS surface modification in PDMS composites.

## 3. Materials and Methods

### 3.1. Chemicals

Commercial two-part vinyl-terminated polydimethylsiloxane (PDMS) (Endeavour T-2516, Endeavour Enterprise Co., Taiwan), whose properties are summarized in Table 2, was used as supplied by the manufacturer. This PDMS is a two-part (A:B) room-temperature-vulcanizing rubber, catalyzed by a platinum complex. Platinum complex participated in a reaction between a hydride functional siloxane polymer and a vinyl functional siloxane polymer, resulting in an ethyl bridge between the two polymers [65]. Hexamethyldisilazane (C_6_H_19_NSi_2_, HMDS, ≥99%, Sigma-Aldrich, Saint Louis, MO, USA) was used as received. The approximate molarity of HMDS (≈4.8 mol L^−1^) was calculated based on its density (0.77 g mL^−1^) and molar mass (161.38 g mol^−1^).

### 3.2. Sample Materials and Preparation

Agro-industrial by-products, namely beetroot (*Beta vulgaris*) peel and pomace, along with sea buckthorn (*Hippophae rhamnoides*), raspberry (*Rubus idaeus*), and shadbush (*Amelanchier* spp.) processing residues, were collected from the production facilities of the Institute of Horticulture, Lithuanian Research Centre for Agriculture and Forestry (LAMMC), Lithuania. The samples were lyophilized using a Zirbus lyophilizer (Zirbus Technology GmbH, Bad Grund, Germany) at 0.01 mbar pressure and a condenser temperature of −85 °C for 3 days. The freeze-dried samples were ground to a powder (particle size 0.2 mm) using a Retsch 200 knife mill (Haan, Germany) and stored in a sealed container for further analysis [66]. Additional physical characteristics of the untreated and HMDS-treated fruit and vegetable by-product powders, including moisture content values, are provided in the Supplementary Materials (Table S5). Oil from raspberry powder and sea buckthorn was also removed by adding hexane to the powder and incubating for 24 h at room temperature in the dark to prevent oxidation. After hexane was removed, the powder was collected. The defatted powders were dried in a ventilated oven at 40–50 °C until complete removal of residual hexane was achieved [67].

### 3.3. Surface Modifications of Fruits and Vegetables By-Product Powders

Surface modification of fruits and vegetable powders was carried out using a simple chemical vapor deposition approach based on the method reported by V. Jankauskaitė et al. [21], with slight modifications. Briefly, approximately 10 g of dried powder was evenly spread on porous filter paper and placed inside a sealed desiccator containing an open vessel with ~10 mL of HMDS. The desiccator was maintained at 65 °C for 24–48 h to allow HMDS vapor to react with the powder surface. This treatment promotes the substitution of surface hydroxyl groups with trimethylsilyl groups, thereby reducing surface polarity and enhancing compatibility with hydrophobic polymer matrices [19,21].

### 3.4. Preparation of Composites

PDMS-based composites incorporating untreated and surface-modified fruit and vegetable by-product powders, as shown in Table 3, were prepared following the methodology reported in V. Jankauskaitė et al., with modifications based on the polymer system described in [21]. Briefly, the PDMS prepolymer Part A and curing agent (Part B) were first mixed, after which the fillers were gradually introduced. The mixtures were manually stirred using a glass stirrer in disposable cups for approximately 1 min to obtain a homogeneous dispersion before being poured into glass Petri dishes for curing. The resulting PDMS/F&V by-product composite mixtures were subsequently degassed under vacuum to remove entrapped air bubbles and then cured at 65 °C for 30 min. Initially, PDMS composites containing 5, 10, 15, and 20 wt.% filler loadings were prepared. Based on preliminary observations, the 5 wt.% and 20 wt.% composites were selected for detailed characterization, and their compositions are presented in Table 3. Composites containing 5 wt.% and 20 wt.% filler loadings were prepared, and their detailed compositions are presented in Table 3. The selected filler loadings represent relatively low (5 wt.%) and high (20 wt.%) filler contents, allowing a comparative evaluation of the influence of filler concentration on the surface, morphological, and mechanical properties of the PDMS composites. During the curing process, a platinum-based catalyst facilitated the hydrosilylation reaction between Si–H groups and vinyl functionalities, leading to the formation of Si–CH_2_–CH_2_–Si crosslinks. This reaction between the PDMS prepolymer and crosslinker chains resulted in the formation of a three-dimensional network.

### 3.5. Color Analysis

PDMS composites incorporated with natural pigments, before and after surface modification, were evaluated following the method of [68] using a MiniScan XE Plus spectrophotometer (Hunter Associates Laboratory, Inc., Reston, VA, USA). Briefly, the apparatus (45/0 geometry, illuminant D65, 10° observer) was calibrated using a standard tile (X = 81.3, Y = 86.2, Z = 92.7). Color indices (L*, a*, and b*) were measured according to the following criteria: L* = 0 represents darkness and L* = 100 represents lightness; +a* = red, −a* = green; +b* = yellow, and −b* = blue.

### 3.6. Measurement of Droplet Contact Angle

The contact angle between a water droplet and the PDMS surface was determined using the sessile drop method, in which a droplet of distilled water was carefully deposited onto the surface of the composites, and the contact angle was measured immediately after stabilization [21]. Briefly, a 10 µL droplet of deionized water was placed on the PDMS surface using a pipette. Contact angle images were captured with a Canon 6D camera (Tokyo, Japan) equipped with a Canon 100 mm macro lens. The contact angles were then analyzed and measured using ImageJ software.

### 3.7. Mechanical Behavior Under Compression

Mechanical behavior was evaluated using a puncture test performed with a texture analyzer (TA.XTplus, Stable Micro Systems, Godalming, UK). The analysis was performed to determine the puncture force, elasticity, and adhesiveness of the samples. Prior to testing, the instrument was calibrated, and a pre-test stage was performed at 5 mm/min until a trigger force of 1 N was reached. Subsequently, the probe penetrated the sample at a constant speed of 500 mm/min. Force–distance data were recorded throughout the test, and the maximum force was defined as the puncture force. Elasticity and adhesiveness were calculated from the force–distance curves using the instrument software. All measurements were performed in triplicate [69].

### 3.8. Morphological and Structural Analysis by EDS-SEM

The morphology and elemental composition of PDMS composites containing plant-based powders were analyzed using a scanning electron microscope (Hitachi S-3400N, Hitachi High-Tech, Tokyo, Japan) equipped with an EDS detector (Bruker Quad 5040, Bremen, Germany) at the Lithuanian Energy Institute (Kaunas, Lithuania). Fractured composite samples were mounted on aluminum stubs with carbon tape and sputter-coated with a thin conductive layer of gold or carbon. SEM imaging was performed under high-vacuum conditions at accelerating voltages of 5–20 kV using secondary and backscattered electron modes to evaluate filler dispersion and interfacial features. EDS spectra and elemental maps were acquired from selected regions of the composite cross-sections to determine elemental composition and distribution using standardless quantification [70].

### 3.9. Fourier-Transform Infrared Spectroscopy FTIR Analysis

FTIR spectra of plant powder samples were recorded using a Bruker Tensor 27 Fourier transform infrared spectrometer (continuous operation process gas analyzer FT-IR spectrometer, Lithuanian Energy Institute, Kaunas, Lithuania). Plant powders were analyzed in their solid form using the attenuated total reflectance ATR accessory directly, without further chemical treatment. Spectra were collected at room temperature over the mid-infrared region in the range of 4000–400 cm^−1^, with a resolution of 4 cm^−1^, and averaged over multiple scans to improve the signal-to-noise ratio. The resulting spectra were processed to identify characteristic vibrational bands corresponding to functional groups present in the plant material [68].

### 3.10. Statistical Analysis

All measurements were performed in triplicate and expressed as mean ± standard deviation. Statistical analysis was carried out using one-way analysis of variance (ANOVA), followed by Tukey’s honestly significant difference (HSD) test, to determine significant differences between means at *p* < 0.05 using Statistic 8.1 and RStudio (Posit Software, PBC, Boston, MA, USA; version 2025.05.1+513) [71].

## 4. Conclusions

This study demonstrates that vapor-phase hexamethyldisilazane (HMDS) treatment can effectively enhance the compatibility of pigment-rich agro-industrial by-product powders with hydrophobic polydimethylsiloxane (PDMS) matrices. FTIR analysis confirmed successful surface modification of sea buckthorn, shadbush, beetroot, and raspberry powders by showing reduced hydroxyl signals and stronger Si-containing bands, which indicate decreased surface polarity after silylation.

Scanning electron microscopy (SEM) observations suggested that HMDS treatment increased powder surface roughness and promoted improved particle–matrix interactions, with fewer visible interfacial gaps in the composites. Contact angle measurements confirmed that surface modification enhanced composite hydrophobicity, with the highest contact angles observed in HMDS-treated composites at 20 wt.% filler loading.

Mechanical testing indicated that filler loading and surface modification significantly influenced composite performance. Composites containing 5 wt.% HMDS-treated fillers, particularly raspberry and sea buckthorn powders, exhibited improved puncture resistance and elasticity compared with untreated fillers. In contrast, increasing filler content to 20 wt.% generally reduced mechanical performance, likely due to particle agglomeration and reduced matrix continuity.

Overall, the results demonstrate that HMDS vapor-phase treatment effectively tailors the filler–matrix interface, enhancing hydrophobicity, filler dispersion, and selected mechanical properties of PDMS composites containing agro-industrial by-product fillers. These findings highlight the potential of pigment-rich fruit and vegetable by-products as functional fillers and natural colorant sources for silicone-based composite materials.

Future studies should include more comprehensive physicochemical characterization of the agro-industrial by-product fillers and evaluate the long-term stability, including color stability during prolonged exposure to air and relevant environmental conditions, to further elucidate the relationships between filler characteristics, HMDS surface modification, and composite performance.

The effects of HMDS treatment were found to be filler-dependent, with improvements observed for some fillers but not for all compositions. Future studies should include standard tensile testing and comparisons with neat PDMS to further evaluate the influence of filler type and loading on composite performance.

## Figures and Tables

**Table 1:** Mechanical properties of polydimethylsiloxane PDMS composites reinforced with raw and HMDS-treated pigment-rich agro-waste fillers. Values are expressed as mean ± standard deviation (SD, *n* = 3). Different superscript letters within each column indicate significant differences among treatments (*p* < 0.05) according to Tukey’s honestly significant difference (HSD) test. For adhesiveness, no significant differences were observed; therefore, all values share the same superscript letter.

Composite		Puncture Force (N·cm^−2^)	Elasticity (N·cm^−2^·sec^−1^)	Adhesiveness (N·cm^−2^)
**Raspberry**	5 wt.% PDMS	200.42 ± 12.9 ^bc^	12.04 ± 1.1 ^ef^	−24.89 ± 3.4 ^a^
	20 wt.% PDMS	135.49 ± 4.7 ^d^	13.62 ± 0.9 ^de^	−24.19 ± 2.4 ^a^
	5 wt.% PDMS-HMDS	252.78 ± 1.8 ^a^	19.22 ± 0.9 ^a^	−29.90 ± 1.7 ^a^
	20 wt.% PDMS-HMDS	133.22 ± 5.5 ^d^	14.09 ± 0.6 ^cd^	−21.42 ± 1.8 ^a^
**Beetroot**	5 wt.% PDMS-HMDS	212.12 ± 16.1 ^b^	13.09 ± 0.1 ^def^	−26.80 ± 2.6 ^a^
	20 wt.% PDMS-HMDS	179.95 ± 5.2 ^c^	15.85 ± 0.3 ^bc^	−24.25 ± 1.5 ^a^
	5 wt.% PDMS	252.99 ± 23.9 ^a^	16.60 ± 0.5 ^b^	−30.06 ± 2.8 ^a^
	20 wt.% PDMS	216.09 ± 14.5 ^b^	17.80 ± 1.1 ^ab^	−20.24 ± 16.1 ^a^
**Sea buckthorn**	5 wt.% PDMS	245.02 ± 3.3 ^a^	16.51 ± 0.4 ^b^	−30.27 ± 3.3 ^a^
	20 wt.% PDMS	131.93 ± 2.0 ^d^	13.65 ± 0.3 ^de^	−22.78 ± 0.9 ^a^
	5 wt.% PDMS-HMDS	251.43 ± 6.2 ^a^	16.92 ± 0.4 ^b^	−23.34 ± 15.0 ^a^
	20 wt.% PDMS-HMDS	130.92 ± 1.6 ^d^	14.13 ± 1.0 ^cd^	−19.10 ± 0.7 ^a^
**Shadbush**	5 wt.% PDMS-HMDS	93.43 ± 7.7 ^e^	7.28 ± 0.5 ^i^	−19.06 ± 2.0 ^a^
	20 wt.% PDMS-HMDS	85.10 ± 3.1 ^e^	9.02 ± 0.3 ^hi^	−16.37 ± 0.6 ^a^
	5 wt.% PDMS	200.72 ± 5.4 ^bc^	13.51 ± 0.2 ^de^	−28.12 ± 1.1 ^a^
	20 wt.% PDMS	99.34 ± 3.6 ^e^	9.93 ± 0.2 ^gh^	−20.50 ± 1.3 ^a^

**Table 2:** Properties of Polydimethylsiloxane PDMS.

Property	Units	Value
**Viscosity (A: B = 1:1, 25 °C)**	Pa·s	3.5
**Hardness**	Shore A	10
**Thermal conductivity**	W/(m·K)	0.2
**Dielectric strength**	kV/mm	27
**Dielectric constant (25 °C, 100 kHz)**	–	4.5
**Volume resistivity**	Ω·m	2.2 × 10^16^

**Table 3:** Composition of PDMS composites containing untreated and HMDS-treated pigment-rich agro-industrial by-product fillers.

Filler Loading (% *w*/*w*)	Raspberry	Sea Buckthorn	Shadbush	Beetroot
**5 wt.%**	Raw powder	Raw powder	Raw powder	Raw powder
**5 wt.%**	Surface-modified powder	Surface-modified powder	Surface-modified powder	Surface-modified powder
**20 wt.%**	Raw powder	Raw powder	Raw powder	Raw powder
**20 wt.%**	Surface-modified powder	Surface-modified powder	Surface-modified powder	Surface-modified powder

## Data Availability

The original contributions presented in this study are included in the article/Supplementary Materials. Further inquiries can be directed to the corresponding authors.

## Supplementary Materials

The following supporting information can be downloaded at: https://www.mdpi.com/article/10.3390/molecules31142546/s1.
