# Supplementary material for: Comparative Study of Raw and HMDS-Treated Pigment-Rich Agro-Industrial By-Products as Functional Fillers in PDMS Composites
Source: Molecules. 2026 Jul 22;31(14):2546. doi: 10.3390/molecules31142546 (PMC13415652; doi:10.3390/molecules31142546)
Supplement: Supplementary file 1 [file molecules-31-02546-s001.zip › molecules-4416648-supplementary.pdf]

Table S1. Quantitative EDS analysis showing the elemental composition (weight percentage and atomic percentage) of HMDS-treated beetroot powder.

Element	Series	unn. C [wt. %]	norm. C [wt. %]	Atom. C [at. %]	Error [%]
Carbon	K-series	38.09	38.09	46.05	16.1
Oxygen	K-series	57.27	57.27	51.98	18.2
Potassium	K-series	2.84	2.84	1.05	0.1
Aluminium	K-series	0.34	0.34	0.18	0.0
Silicon	K-series	0.26	0.26	0.13	0.0
Sodium	K-series	0.21	0.21	0.13	0.0
Chlorine	K-series	0.42	0.42	0.17	0.0
Phosphorus	K-series	0.21	0.21	0.10	0.0
Magnesium	K-series	0.18	0.18	0.11	0.0
Sulfur	K-series	0.18	0.18	0.08	0.0

Table S2. Quantitative EDS analysis showing the elemental composition (weight percentage and atomic percentage) of HMDS-treated raspberry powder.

Element	Series	unn. C [wt. %]	norm. C [wt. %]	Atom. C [at. %]	Error [%]
Carbon	K-series	44.69	44.69	52.50	16.7
Oxygen	K-series	52.40	52.40	46.21	16.9
Potassium	K-series	0.68	0.68	0.24	0.1
Calcium	K-series	0.53	0.53	0.19	0.1
Magnesium	K-series	0.34	0.34	0.20	0.1
Aluminium	K-series	0.45	0.45	0.24	0.1
Silicon	K-series	0.25	0.25	0.13	0.1
Phosphorus	K-series	0.36	0.36	0.16	0.0
Sulfur	K-series	0.30	0.30	0.13	0.0

Table S3. Quantitative EDS analysis showing the elemental composition (weight percentage and atomic percentage) of HMDS-treated sea buckthorn powder.

Element	Series	unn. C [wt. %]	norm. C [wt. %]	Atom. C [at. %]	Error [%]
Carbon	K-series	45.47	45.47	53.17	19.9
Oxygen	K-series	52.36	52.36	45.96	16.6
Potassium	K-series	0.92	0.92	0.33	0.1
Phosphorus	K-series	0.26	0.26	0.12	0.0
Aluminium	K-series	0.25	0.25	0.13	0.0
Silicon	K-series	0.11	0.11	0.05	0.0
Sulfur	K-series	0.18	0.18	0.08	0.0
Chlorine	K-series	0.17	0.17	0.07	0.0
Calcium	K-series	0.28	0.28	0.10	0.0

Table S4. Quantitative EDS analysis showing the elemental composition (weight percentage and atomic percentage) of HMDS-treated shadbush powder.

Element	Series	unn. C [wt. %]	norm. C [wt. %]	Atom. C [at. %]	Error [%]
Carbon	K-series	44.48	44.49	52.25	16.9
Oxygen	K-series	52.94	52.95	46.68	17.1
Potassium	K-series	1.03	1.03	0.37	0.1
Calcium	K-series	0.60	0.60	0.21	0.1
Aluminium	K-series	0.28	0.28	0.15	0.0
Silicon	K-series	0.16	0.16	0.08	0.0
Phosphorus	K-series	0.26	0.26	0.12	0.0
Magnesium	K-series	0.24	0.24	0.14	0.0

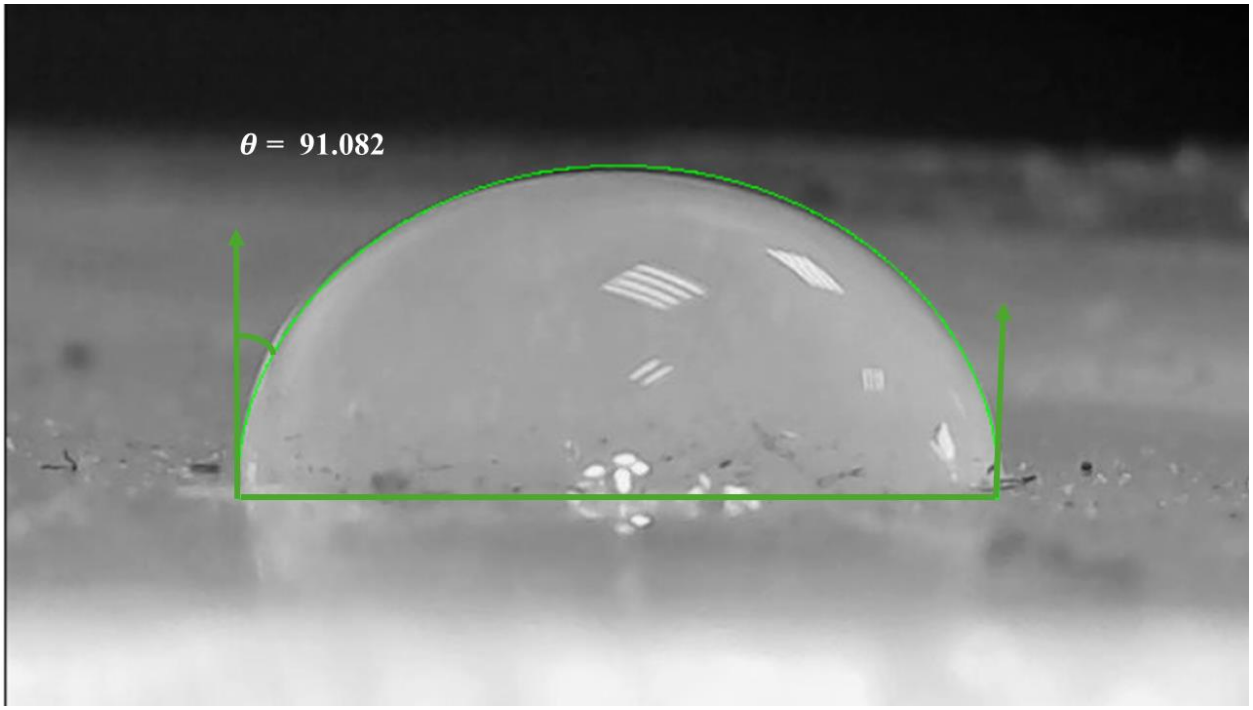

Figure S1. Water contact angle measurement of neat PDMS showing its intrinsic hydrophobic nature (contact angle = 91.08°).

Table S5. Moisture content of untreated and HMDS-treated fruit and vegetable by-product powders.

Fruits and vegetable By-product powder	Moisture content of Untreated Fruits and vegetabl By-product powder (db%)	Moisture content of HMDS treated Fruits and vegetable By-product powder (db%)
Raspberry	92.18	90.51
Beetroot	89.38	92.41
Sea buckthorn	92.68	92.04
Shadbush	95.49	99.60
